# Dihydroosajaxanthone: A New Natural Xanthone from the Branches of *Garcinia Schomburgkian*a Pierre

**Published:** 2018

**Authors:** Imron Meechai, Worrapong Phupong, Warangkana Chunglok, Puttinan Meepowpan

**Affiliations:** a *Department of Chemistry, School of Science, Walailak University, Nakhon Si Thammarat, 80161, Thailand. *; b *Department of Medical Technology, School of Allied Health Sciences, Walailak University, Nakhon Si Thammarat, 80161 Thailand. *; c *Department of Chemistry, Faculty of Science, Chiang Mai University, Chiang Mai, 50200, Thailand.*

**Keywords:** Dihydroosajaxanthone, Garcinia schomburgkiana, Clusiaceae, Xanthone, Phytochemical

## Abstract

*Garcinia schomburgkiana*, locally known in Thailand as an edible fruit “Ma-dan”, is a plant species of the Clusiaceae family which has been reported as sources of a variety of compounds with biological activities. In the phytochemical studies of Ma-dan, four xanthones were, for the very first time, isolated from the branch acetone extract of *G. schomburgkiana*. Their structures were determined through the analysis of spectroscopic data (^1^H, ^13^C-NMR, IR and MS) and the comparison with those previously reported. Dihydroosajaxanthone (**1**), an original synthetic xanthone, is reported herein for the first time as a naturally occurring xanthone, together with three known xanthones: xanthochymone A (**2**), 1,3,7-trihydroxy-2-(3-hydroxy-3-methylbutyl) xanthone (**3**) and 1,3,5,6-tetrahydroxyxanthone (**4**). These compounds, especially dihydroosajaxanthone (**1**), might be considered as chemotaxonomic markers of the Garcinia genus.

## Introduction

*Garcinia schomburgkiana* Pierre. is an edible plant in the Clusiaceae family, known in Thai as Ma-dan. It has been traditionally used as a cough treatment, a diabetes medication and a laxative (1). The Garcinia species has been widely studied on their chemical constituents and biological activities (2). The species has been reported as rich sources of xanthones (3), which are normally found in higher plants. The phytochemical investigation of the wood, the stem and the bark of *G. schomburgkiana* led to the isolation of xanthones, benzophenones, biphenyl compounds and biflavonoids (4-7). However, to the best of our knowledge, there is not yet any report on the constituents from the branches*.*

## Experimental

*General methods*

The ^1^H and ^13^C nuclear magnetic resonance (NMR) spectra were recorded on Varian Unity Inova 500 MHz spectrometer in dimethyl sulfoxide-d_6_ and acetone-d_6_ as solvents. The FT-IR spectra were recorded on a Bruker Tensor 27 spectrophotometer. The HR-ESIMS were carried out on a Bruker microTOF-Q spectrometer. A column chromatography (CC) was run on silica gel (SiO_2_, Merck, 40-63 *µ*m), and Sephadex LH-20 (GE Healthcare). The thin-layer chromatography (TLC) analysis was performed on silica gel (SiO_2_, Merck, 60 F_254_), visualized under the UV light at 254 or 366 nm and stained with the *p*-anisaldehyde solution in 2% H_2_SO_4_/EtOH. All solvents, used for extraction and isolation, were distilled at their boiling point ranges prior to use.

*Plant material*

In this study, *G. schomburgkiana* branches were collected from the Yan Ta Khao district, Trang Province, Thailand and the voucher specimen (GS-001WU) is deposited at the Research Unit of Natural Product Utilization, Walailak University.

*Extraction and isolation*

**Figure 1:**
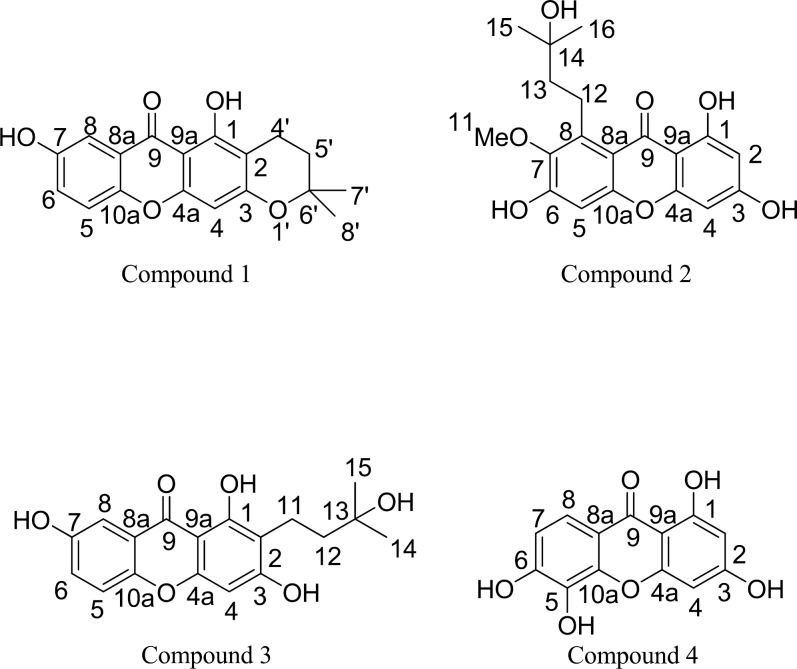
The structure of compound **1-4**

The phytochemicals (Figure 1) were isolated from the branches of *G. schomburgkiana *according to the following protocols*.* The air-dried materials (17.5 kg) were macerated two times with acetone (32 L) for 5 days at room temperature. The extracts were concentrated under vacuum to give crude extract (400 g). The acetone extract (100 g) was then chromatographed on a silica gel column, eluted with a gradient of CH_2_Cl_2_: MeOH, to yield 12 fractions (A-L). Fraction F (8.72 g) was separated by the column chromatography (silica gel, hexane: EtOAc, 90:10 to 0:100) to obtain 6 sub-fractions (F1-F6). Sub-fraction F3 (3.85 g) was isolated by the column chromatography (silica gel, hexane: EtOAc, 75:25 to 0:100) to provide 5 sub-fractions (F3A-F3E). Compound **1** (9.5 mg) was from the purification of sub-fraction F3B (851.8 mg) through the sephadex LH-20 column chromatography (MeOH: CH_2_Cl_2, _50:50). For the isolation of compound **2** and **3**, Fraction H (6.44 g) was partitioned by the column chromatography (silica gel, CH_2_Cl_2_: MeOH, 100:0 to 80:20) to afford 8 sub-fractions (H1-H8). Sub-fraction H5 (1.49 g) was separated by the sephadex LH-20 column chromatography, eluted with 50% MeOH in CH_2_Cl_2_, to give 12 sub-fractions (H5A-H5L). Sub-fractions H5E (103.1 mg) was further isolated by the column chromatography (silica gel, CH_2_Cl_2_: MeOH, 95:5 to 80:20) to afford 5 sub-fractions (H5E1-H5E9). Compound 2 (5.0 mg) and compound **3** (2.8 mg) were obtained by the silica gel column chromatography (CH_2_Cl_2_: MeOH 95:5) of sub-fractions H5E (37.6 mg) and sub-fractions H5F (35.7 mg), respectively. To isolate compound **4**, Fraction J (26.65 g) was separated by the column chromatography (silica gel, CH_2_Cl_2_: MeOH, 80:20 to 50:50) to afford 6 sub-fractions (J1-J6). Sub-fractions J2 (1.95 g) was isolated by the sephadex LH-20 column chromatography, eluted with 100% MeOH, to give 10 sub-fractions (J2A-J2J). Sub-fractions J2D (154.9 mg) was further purified by the column chromatography (silica gel, CH_2_Cl_2_: MeOH, 90:10) to afford compound** 4** (5.1 mg).

Compound **1**: Needle yellow crystal; IR (neat) *υ*_max_ cm^-1^: 3384 (OH, hydroxyl), 1640 (C=O, carbonyl), 1582 (C=C, aromatic); C_18_H_16_O_5_, HR-ESIMS [M+Na]^+^ 335.0894 m/z: (calcd. for C_18_H_16_O_5_Na 335.0895); NMR: see Table 1.

Compound **2**: Yellow powder; IR (neat) *υ*_max_ cm^-1^: 3389 (OH, hydroxyl), 1647 (C=O, carbonyl), 1589 (C=C, aromatic); C_19_H_20_O_7_, HR-ESIMS [M+K]^+^ 399.0850 m/z: (calcd. for C_19_H_20_O_7_K 399.0846); NMR: see Table 2.

Compound **3**: Yellow powder; IR (neat) *υ*_max_ cm^-1^: 3419 (OH, hydroxyl), 1647 (C=O, carbonyl), 1580 (C=C, aromatic); C_18_H_18_O_6_, HR-ESIMS [M+K]^+^ 369.0741 m/z: (calcd. for C_18_H_18_O_6_K 369.0740); NMR: see Table 3.

Compound **4**: Yellow powder; IR (neat) υ_max_ cm^-1^: 3190 (OH, hydroxyl), 1636 (C=O, carbonyl), 1513 (C=C, aromatic); C_13_H_8_O_6_, HR-ESIMS [M+H]^+^ 261.0394 m/z: (calcd. for C_13_H_9_O_6_ 261.0399); NMR: see Table 4.

**Table 1:** ^1^H NMR (500 MHz) and ^13^C NMR (125 MHz) data of compound **1 **in dimethyl sulfoxide-d_6_

**6**	***δ***_H_ ** (m, J = Hz)**	***δ***_C_ ** (m)** a	**HMBC**
1 2 3 4 4^a^ 5 6 7 8 8^a^ 9 9^a^ 10^a^ 4’ 5’ 6’ 7’ 8’ 1-OH 7-OH	- - - 6.35 (s) - 7.41 (d, 9.0) 7.27 (dd, 3.0 and 9.0) - 7.44 (d, 3.0) - - - - 2.62 (t) 1.82 (t) - 1.32 (s) 1.32 (s) 13.25 (s) 9.94 (s)	159.8 (s) 103.7 (s) 161.3 (s) 94.4 (d) 155.1 (s) 119.0 (d) 124.7 (d) 153.9 (s) 108.1 (d) 120.4 (s) 180.1 (s) 101.9 (s) 149.2 (s) 15.7 (t) 31.0 (t) 76.7 (s) 26.5 (q) 26.5 (q) - -	- - - C-2, 3, 4^a^, 9^a^ - C-5, 7 C-8, 10^a^ - C-6, 9, 10^a^ - - - - C-2, 3, 5’, 6’ C-3, 4’, 6’, 7’, 8’ - C-4’, 6’, 8’ C-4’, 6’, 7’ C-1, 2, 9^a^ C-6, 8

a Multiplicity was determined by DEPT experiments (s = quaternary, d = methine, t = methylene, q = methyl).

**Table 2:** ^1^H NMR (500 MHz) and ^13^C NMR (125 MHz) data of compound **2 **in acetone-d_6_.

**Position**	***δ***_H_ ** (m, J = Hz)**	***δ***_C_ ** (m)** a	**HMBC**
1 2 3 4 4^a^ 5 6 7 8 8^a^ 9 9^a^ 10^a^ 11 12 13 14 15 16 1-OH	- 6.18 (d, 2.0) - 6.30 (d, 2.0) - 6.82 (s) - - - - - - - 3.84 (s) 3.44 (m) 1.74 (m) - 1.29 (s) 1.29 (s) 13.51 (s)	164.8 (s) 98.6 (d) 165.9 (s) 93.77 (d) 157.6 (s) 102.5 (d) 156.3 (s) 144.5 (s) 140.0 (s) 111.8 (s) 182.7 (s) 103.6 (s) 157.9 (s) 61.52 (q) 23.1 (t) 45.6 (t) 70.0 (s) 29.3 (q) 29.3 (q) -	- C-1, 2, 4, 9^a^ - C-2, 3, 4^a^, 9^a^ - C-7, 9, 8^a^, 10^a^ - - - - - - - C-7 C-7, 8^a^, 13, 14 C-8, 12, 14, 15, 16 - C-13, 14, 16 C-13, 14, 15 C-1, 2, 9^a^

a Multiplicity was determined by DEPT experiments (s = quaternary, d = methine, t = methylene, q = methyl).

**Table 3:** ^1^H NMR (500 MHz) and ^13^C NMR (125 MHz) data of compound **3 **in acetone-d_6_.

**Position**	***δ***_H_ ** (m, J = Hz)**	***δ***_C_ ** (m)** a	**HMBC**
1 2 3 4 4^a^ 5 6 7 8 8^a^ 9 9^a^ 10^a^ 11 12 13 14 15 1-OH	- - - 6.45 (s) - 7.40 (d, 9.0) 7.32 (dd, 3.0 and 9.0) - 7.56 (d, 3.0) - - - - 2.76 (m) 1.70 (m) - 1.24 (s) 1.24 (s) 13.25 (s)	161.3 (s) 112.4 (s) 164.6 (s) 94.1 (d) 156.7 (s) 119.6 (d) 124.8 (d) 154.6 (s) 109.2 (d) 121.8 (s) 181.0 (s) 103.1 (s) 150.6 (s) 17.8 (t) 43.1 (t) 70.5 (s) 29.3 (q) 29.3 (q) -	- - - C-2, 3, 4^a^, 9^a^ - C-7, 8^a^, 10^a^ C-7, 10^a^ - C-6, 9, 10^a^ - - - - C-1, 2, 3, 12, 13 C-2, 13, 14, 15 - C-12, 13, 15 C-12, 13, 14 C-1, 2, 9^a^

a Multiplicity was determined by DEPT experiments (s = quaternary, d = methine, t = methylene, q = methyl).

**Table 4:** ^1^H NMR (500 MHz) and ^13^C NMR (125 MHz) data of compound **4 **in acetone-d_6_

**Position**	***δ***_H_ ** (m, J = Hz)**	***δ***_C_ ** (m)** a	**HMBC**
1 2 3 4 4^a^ 5 6 7 8 8^a^ 9 9^a^ 10^a^ 1-OH	- 6.42 (d, 1.7) - 6.22 (d, 1.7) - - - 6.96 (d, 9.0) 7.60 (d, 9.0) - - - - 13.16 (s)	164.7 (s) 94.6 (s) 165.8 (s) 98.7 (d) 158.6 (s) 133.2 (s) 152.2 (s) 113.6 (d) 117.2 (d) 114.6 (s) 181.0 (s) 103.0 (s) 146.8 (s) -	- C-3, 4, 4^a^, 9^a^ - C-2, 3, 4^a^, 9^a^ - - - C-5, 6, 8^a^ C-6, 9, 10^a^ - - - - C-1, 2, 9^a^

a Multiplicity was determined by DEPT experiments (s = quaternary, d = methine, t = methylene, q = methyl).

## Results and Discussion

The phytochemical study led to the first isolation of four known xanthones from the Ma-dan branch acetone extract. Compound **1**, with a molecular formula C_18_H_16_O_5_ (HR-ESIMS: 335.0894 m/z), showed the IR absorption band for the hydroxyl, carbonyl, and aromatic groups. The ^1^H-NMR data exhibited the characteristic of xanthone with signals of chelated hydroxyl proton at *δ *13.25 (s, 1-OH) and *δ *9.94 (s, 7-OH), a tri-substituted aromatic proton with ABX system at *δ *7.44 (d, J = 3.0, H-8), *δ *7.27 (dd, J = 3.0 and 9.0, H-5) and *δ *7.41 (d, J = 9.0, H-6) and one singlet aromatic proton at *δ *6.35. The characteristic of dihydropyran ring displayed signals at *δ *2.62 (t, H-4’), *δ *1.82 (t, H-5’), and *δ *1.32 (s, H-7’ and H-8’). The ^13^C-NMR data presented eighteen carbons including one quaternary of carbonyl carbon (*δ *180.1, C-9), nine quaternary (*δ *159.8, C-1; *δ *103.7, C-2; *δ *161.3, C-3; *δ *155.1, C-4a; *δ *153.9, C-7; *δ *120.4, C-8a; *δ *101.9, C-9a; *δ *149.2, C-10a and *δ *76.7, C-6’), four methine (*δ *94.4, C-4; *δ *119.0, C-5; *δ *124.7, C-6 and *δ *108.1, C-8), two methylene (*δ *15.7, C-4’ and *δ *31.0, C-5’) and two methyl (*δ *26.5, C-7’ and C-8’). The HMBC correlation of aromatic protons and carbons suggested that the structure of compound 1 was the 1, 3, 7- trioxygenated xanthone fused with a dihydropyran ring at C-2 and C-4. A comparison of the spectra data with those of synthetic compound in literature (8-11) showed that compound 1 was deduced as dihydrosajaxanthone, which is, for the first time, reported as a natural product. Along with dihydrosajaxanthone (**1**), xanthochymone A (**2**) (12), 1,3,7-trihydroxy-2-(3-hydroxy-3-methylbutyl) xanyhone (**3**) (13) and 1,3,5,6-tetrahydroxyxanthone (**4**) (14) (Figure 1) were identified by the analysis of spectroscopic data and comparisons with literatures. Although compound **2-4** were known as naturally occurring xanthones, they were firstly reported herein from this plant. These compounds, especially dihydroosajaxanthone (**1**), might be considered as significant chemotaxonomic makers.
